# A Case of Poisoning by Arsenic—Spontaneous Cure—after Symptoms Removed by the Hydrated Peroxide of Iron

**Published:** 1840-04-18

**Authors:** W. W. Gerhard

**Affiliations:** Attending Physician


### CLINICAL REPORTS.

### PHILADELPHIA HOSPITAL.

*A case of Poisoning by Arsenic—Spontaneous cure—After symptoms removed by the Hydrated Peroxide of Iron.* W. W. Gerhard, M. D., Attending Physician.

[Reported by A. D. Chaloner, M. D.]

Hugh Donnelly, labourer, æt. 44 years, admitted April 7th, 1840, at 3 P. M. Was in good health on the 6th April; was intoxicated for three or four days, before had been tolerably sober. Having been robbed of $1300 on the evening of the 6th inst., at eight o'clock he took, with the intention of committing suicide, a portion of arsenic (about *half an ounce*, or six and a quarter cents worth) in a wineglass of gin.

In three quarters of an hour afterwards, he felt bad, with great thirst and vomiting; pain at the epigastrium and abdomen, (this, in about an hour, "*soon sobered him;*") purging came on at the same time with the vomiting; he vomited incessantly until about twelve o'clock at night, when he felt comparative ease during rest, and at times until twelve o'clock the next day.

Entered the Philadelphia Hospital at three, P. M., (April 7th,) felt very weak, soreness of throat and retching, which lasted after his entrance; the purging was accompanied by a sensation of heat.

A quarter of an hour after he took some oil, and the *hydrated peroxide of iron* FREELY, which last seemed to check the vomiting. Before his entrance, took nothing but abundant draughts of water with some eggs broken up in it.

Afterwards, for two days, the pain at the epigastrium was renewed, but there was no more vomiting; has had no pains or soreness in his bones. Took nothing but an occasional dose of oil, and

℞.—Hydrarg. Chlorid. mitis gr. x.
Jalap, gr. xv.

*April* 22.—A recurrence of the soreness and pain in the region of the spleen; was cupped in that region; appetite indifferent; after taking some gruel, had increase of gastric symptoms; still felt weak; blister over the spleen.

*April* 24.—Tongue whitish, now moist, at times quite dry; no pain at epigastrium, excepting a slight soreness; pulse ninety-four, and skin moist. ℞. Ol. Ricini ℥iv. Blister to be kept open.

*April* 27.—Slight soreness at epigastrium; in other respects comfortable; diet gruel.

*Remarks.*—The quantity of arsenic taken in this case was ascertained by inquiring of the apothecary from whom the patient had purchased it, under the plea of poisoning rats. He affirms, positively, that he swallowed the whole quantity. The safety of the patient apparently arose from the free vomiting and purging produced by the arsenic, as the eggs were taken some time after these evacuations.

The patient was seen by Dr. Baker, the resident physician, immediately after his entrance. Dr. B. directed for him the hydrated peroxide of iron in as large quantities as he could take, notwithstanding the previous evacuations. This remedy produced immediate relief, showing that after the urgent symptoms of poisoning by arsenic are passed, the preparation of iron is still of service. An interesting paper by Dr. Fisher upon this preparation of iron, and the means of preserving it for use, was copied into the Examiner from the Journal of Pharmacy.

In most cases of poisoning by arsenic, the substance is purchased from druggists under the pretext of poisoning rats. The apothecary from whom the arsenic was procured in this instance, is one of the most respectable in the city, and although cautious in discriminating the persons to whom he sells so powerful a poison, was completely deceived in this instance. A fatal termination would have been the more lamentable, as the desire to commit suicide was temporary, and ceased with the recovery of the patient from the fit of intoxication.

